# Multifaceted Impact of the COVID-19 Pandemic and Lockdown on Physical and Mental Health: Insights from a Cross-Sectional Study

**DOI:** 10.1192/j.eurpsy.2024.1073

**Published:** 2024-08-27

**Authors:** V. Dimitriou, C. Kalogirou, A. Potamianou, M. Bakola, P. Gourzis, G. Charalampous, E. Jelastopulu

**Affiliations:** ^1^Postgraduate Program Health Management, Frederick University, Nicosia, Cyprus; ^2^Medical School, Department of Public Health; ^3^Medical School, Department of Psychiatry, University of Patras, Patras, Greece

## Abstract

**Introduction:**

The global COVID-19 pandemic and subsequent lockdowns have significantly impacted global wellbeing and highlighted the close link between mental and physical health. Social isolation and quarantine have proven to be major stressors, leading to emotional distress and unpredictable psychological consequences.

**Objectives:**

We explored the pandemic’s impact on individuals’ physical and mental health and social relationships.

**Methods:**

We conducted a cross-sectional study using a questionnaire which included among other socio-democratic questions, the Fear of COVID-19 Scale, the World Health Organization Quality-of-Life Scale (WHOQOL-BREF) and the Toronto Empathy Questionnaire (TEQ).

**Results:**

A total of 511 adults (55.1% males) participated in this study. Participants reported increased social media use (more than 4-5 times/week) during the lockdown, which was associated with increased fear of COVID-19 and negative effects on mental and physical health, and social relationships (p<0.01). Conversely, non-work-related outings (once a week) were associated with lower fear (p<0.01) and better well-being (p<0.05). Higher fear, particularly for loved ones, was associated with negative effects. The level of physical health was moderate to high, with varying levels of satisfaction in different areas. Empathy correlated with increased fear (p<0.01) and reduced mobility (p<0.05).

**Conclusions:**

The COVID-19 pandemic and lockdowns significantly affected physical and mental health, highlighting the importance of tailoring interventions for vulnerable populations and promoting adaptive coping strategies in times of crisis.

**Disclosure of Interest:**

None Declared

